# Cultural adaptation and reliability assessment of the Hammersmith neonatal neurological examination for Brazilian newborns at risk of cerebral palsy

**DOI:** 10.1055/s-0042-1758863

**Published:** 2023-03-14

**Authors:** Mayara Thais Correr, Luzia Iara Pfeifer

**Affiliations:** 1 Universidade de São Paulo, Faculdade de Medicina de Ribeirão Preto, Departamento de Neurociências e Ciências do Comportamento, Programa de Pós-graduação em Neurologia, Ribeirão Preto SP, Brazil.

**Keywords:** Cerebral Palsy, Early Diagnosis, Neurology, Paralisia Cerebral, Diagnóstico Precoce, Neurologia

## Abstract

**Background** Reliable instruments that lead to early diagnosis for CP are extremely important so that these children are referred for early stimulation, benefiting their development.

**Objective** To perform a cross-cultural adaptation and reliability assessment of a Brazilian version of the Hammersmith Neonatal Neurological Examination (HNNE), expanded and summarized.

**Methods** A methodological, cross-sectional, nonexperimental quantitative analysis was conducted in two phases as follows: cultural adaptation of the HNNE, expanded and summarized, and reliability assessment of the Brazilian version of the HNNE. Phase one was developed in five stages (initial translation, synthesis of the translation, a committee of experts, backtranslation, and submission to the author), with the semantic questions, content, and face validity being evaluated. Phase two included 143 newborns and we analyzed the internal consistency, stability, and equivalence (intra- and interexaminer) of the instrument. Internal consistency was calculated using Cronbach's alpha, and intra- and interexaminer reliability and reproducibility assessed through test-retest were calculated using the intraclass correlation coefficient

**Results** Although internal consistency, assessed using Cronbach's alpha, showed unsatisfactory results, the results of inter-and intraexaminer equivalence showed a high agreement between the evaluations in all domains. The test-retest also showed excellent agreement between the domains.

**Conclusions** The Brazilian HNNE expanded and summarized versions can be considered to be adapted and reliable for the neurological assessment of Brazilian newborns to identify changes in neurological development and early referral to the stimulation or early rehabilitation units and as a promising option to be used in the context of primary care in Brazil.

## Resumo

**Antecedentes** As avaliações neurológicas que levam ao diagnóstico precoce permitem o acesso oportuno à intervenção em um período em que os maiores ganhos são possíveis devido à neuroplasticidade.

**Objetivos** Realizar a adaptação transcultural e avaliação da confiabilidade da versão brasileira do Hammersmith Neonatal Neurological Examination (HNNE), ampliada e resumida.

**Métodos** Foi realizada análise quantitativa metodológica, transversal e não experimental em duas fases: adaptação cultural do HNNE, ampliada e resumida, e avaliação da confiabilidade da versão brasileira do HNNE. A primeira fase foi desenvolvida em cinco etapas (tradução inicial, síntese da tradução, comitê de especialistas, retrotradução e submissão ao autor), sendo avaliadas as questões semânticas, conteúdo e validade de face. A fase dois incluiu 143 recém-nascidos e foram analisadas a consistência interna, estabilidade e equivalência (intra e interexaminador) do instrumento. A consistência interna foi calculada pelo alfa de Cronbach, e a confiabilidade e reprodutibilidade intra e interexaminadores avaliadas por meio do teste-reteste foram calculadas pelo coeficiente de correlação intraclasse.

**Resultados** Embora a consistência interna, avaliada pelo alfa de Cronbach, tenha apresentado resultados insatisfatórios, os resultados da equivalência inter e intraexaminadores mostraram alta concordância entre as avaliações em todos os domínios. O teste-reteste também apresentou excelente concordância entre os domínios.

**Conclusões** As versões brasileiras ampliadas e resumidas do HNNE podem ser consideradas adaptadas e confiáveis para avaliação neurológica de recém-nascidos brasileiros por identificar alterações no desenvolvimento neurológico e encaminhamento precoce para unidades de estimulação ou reabilitação precoce e como uma opção promissora para uso no contexto da atenção básica no Brasil.

## INTRODUCTION

Neurological assessment is one of the most widely used clinical tools to monitor the development of babies at risk of neurological disabilities. 1 2 Currently, most scientific studies are linked to the assessment of global development and the acquisition of motor, cognitive, language, and socioemotional skills. 3 However, studies on neurological assessments that lead to early diagnosis are essential, allow timely access to intervention in a period where the greatest gains are possible due to neuroplasticity. Late diagnosis can be detrimental to the development of a child and can deprive them of early intervention for months or even years. 4

The tools with the best predictive validity to detect cerebral palsy (CP) are neonatal magnetic resonance imaging, Prechtl Qualitative Assessment of General Movements, and the Hammersmith Neurological Examination. 1 The Hammersmith Neurological Examination has two versions: the expanded and summarized versions of the Hammersmith Neonatal Neurological Examination (HNNE), which evaluates newborns (NBs) up to 3 months old, and the Hammersmith Infant Neurological Examination (HINE), which evaluates infants between 30 days and 24 months old. 5

The expanded version of the HNNE consists of 34 items subdivided into 6 categories as follows: 1) posture and tone, 2) tone patterns, 3) reflexes, 4) movements, 5) abnormal signs, and 6) orientation and behavior. The examination takes ∼ 15 minutes and can be used to assess NBs with unstable conditions, without the need to follow the sequence proposed in the evaluation form. One can also choose the most appropriate sequence in relation to the positioning of the baby or their alertness. 6 Each item has figures and descriptions and may be scored as 0.0 (abnormal), 0.5 (intermediate), or 1.0 (normal). The total score is calculated as the sum of the individual item scores, with the normal range being 30.5 to 34. If the global score is in the borderline zone, it does not necessarily mean that the assessed NB presents neurological abnormalities, but it identifies that regular neurological follow-up must be maintained. 6

**Figure 1:**
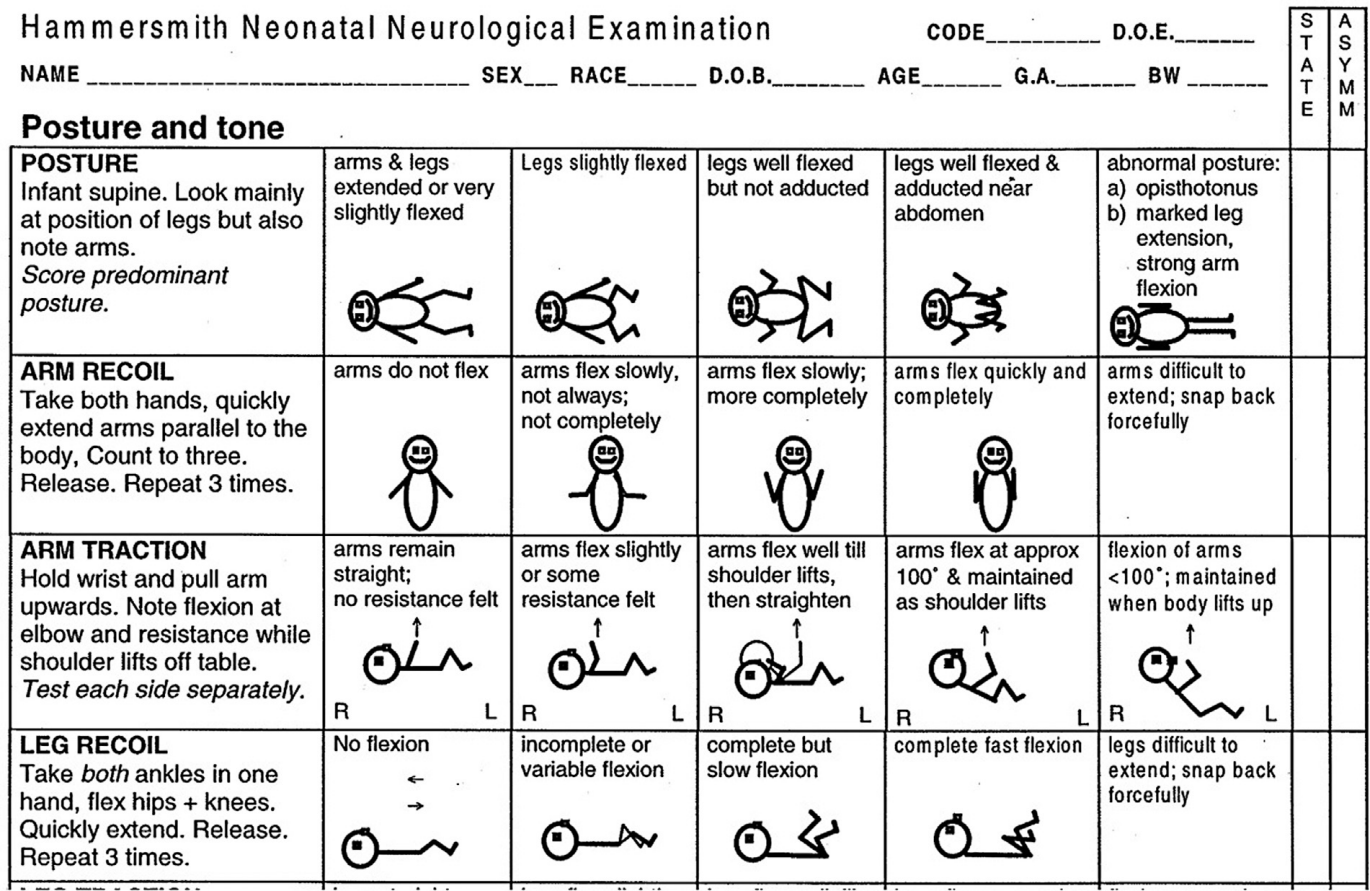
A short and simplified version of the HNNE, the behaviors listed in the first and last columns are abnormal.

In 2005, a short and simplified version of the HNNE was prepared for screening, which consisted of 25 items under the following categories: posture and tone, movements, reflexes, guidance, and behavior. 5 The behaviors listed in the first and last columns are abnormal for term infants ( Figure 1 ); therefore, if two or more items in these columns are scored or one or more of the abnormal signs that are listed at the end of the instrument are noted, then the infant must be evaluated by the full version. 7 Both versions can be applied to premature infants. 6

In Brazil, validated assessment tools for predicting neurological disorders are rare. The HNNE has been identified as one of the best and simplest neurological examinations for the early diagnosis of neurological impairment in low- and high-risk neonates, and it is an easily applied tool, even by inexperienced professionals. 2 8 Although there are already translations in Portuguese from Brazil and Portugal, elaborated by Tathiana Ghisi de Souza e Moyra Aloia Romero (linked in hammersmith-neuro-exam.com), this translation was done freely, without the cross-adaptation process. The cultural adaptation process includes a translation by two independent translators, the committee of experts, synthesis of translations, and backtranslation. 9 10 This way, we decided to perform a cross-cultural adaptation and assess the reliability of a Brazilian version of the HNNE (expanded and summarized).

## METHODS

The present methodological, cross-sectional, nonexperimental study, approved by the ethics committee under opinion No. 1,809,858, was conducted in 2 phases as follows: cultural adaptation of the HNNE (expanded and summarized) and reliability assessment of the Brazilian version of the HNNE (expanded).

### Phase 1

Cultural adaptation of the HNNE - expanded and summarized 6 to Brazilian neonates.

We obtained authorization from the authors of the instrument via email to carry out the HNNE translation, cultural adaptation, and psychometric validation of the expanded and summarized versions. The translation and cultural adaptation procedures followed the guidelines proposed by Wild et al., 9 Beaton et al., 10 Pasquali, 11 and Ferrer et al., 12 in five stages as follows:

#### Stage 1 - initial translation

Two Brazilian translators (T1 and T2) independently translated the original version of the HNNE (expanded and summarized). T1 had knowledge in the field of neuropediatrics and knew the concepts examined by the instrument, while T2 had none of this knowledge. However, both translators had proficiency in both languages (English and Portuguese) and prepared translation versions 1 and 2 (VT1 and VT2), respectively.

#### Stage 2 - synthesis of the translations

A technical committee comprising an experienced researcher in the field of neuropediatrics, a neurology postgraduate, and a doctor-researcher in the field of neuropediatrics with > 25 years of experience, all with proficiency in both languages, was formed to compare VT1 and VT2 and to elaborate the Portuguese synthesized version (VSP). This procedure followed the recommendations made by Koller et al. 13 when we merged VT1 and VT2 with modifications/additions or used VT2 adapted to VT1 and vice-versa.

#### Stage 3 - committee of experts

The original and VSP versions were analyzed for their semantic and content by a committee of experts comprising 10 professionals with experience in neuropediatrics. Face validity was also performed at this stage, since the committee was formed by professionals in the area, to point out clarity in the items that made up the instrument. The technical committee analyzed all suggestions of experts and re-evaluated and restructured all items with < 80% agreement and sent them to experts again until an acceptable deal was reached, as proposed by the literature. 10 11 Thus, the technical committee elaborated on the Portuguese consensual version (VCP), which was then sent for backtranslation.

#### Stage 4 - back-translation

The VCP was backtranslated into English by two independent backtranslators who were native English speakers and had proficiency in Portuguese, both without knowledge of the original version of the instrument and without experience in neuropediatrics. The same technical committee of stage 2 analyzed the semantic, idiomatic, cultural, and conceptual equivalences and produced the synthesis of backtranslations 1 and 2 by elaborating the consensual back-translation version (VCRT). This step was important to validate whether the translation reflected the same content as the items in the original instrument.

#### Stage 5 - submission to the author

The VCP and VCRT versions were sent to the author of the instrument and the team who approved the versions consolidating the final version (HNNE-Br) of the HNNE instrument in all their versions (HNNE expanded and summarized + HINE) for the Brazilian population. It is important to note that the authors did not make suggestions or indicate changes to be made in the documents sent to them.

The expanded HNNE had 34 items, each with 5 possible answers; thus, in the stages of cultural adaptation, it was broken down into 170 topics of analysis, in addition to items that included comments, observations, characterization of the evaluated items, and a summary of the instrument, totaling 214 topics translated and adapted to the Brazilian culture.

The summarized HNNE had 25 items, each with 5 possible answers, and it was broken down, totaling 157 topics translated and adapted to the Brazilian culture.

### Phase 2

Reliability of the Brazilian version of the HNNE (HNNE-Br) – expanded and summarized.

A convenience sample of 143 premature and full-term neonates who presented some risk for CP were recruited from obstetric outpatient, neonatal maternity, and intermediate care units (UCIN), of two hospitals of a city located in the interior of the state of São Paulo, Brazil.

We analyzed the internal consistency, stability, and equivalence to verify the reliability of the HNNE-Br (expanded). The 25 items of the HNNE-Br summarized are present in the expanded version, so the results are the same for both versions:

- The Cronbach alpha test analyzed the internal consistency 14 of the HNNE-Br (expanded) and was performed with a total sample of 143 NBs. The evaluations took place as near as possible after birth, and some NBs were evaluated a few hours after birth, while others a few days later.
- Intra- and interexaminer reliability was analyzed to assess equivalence. The evaluations were filmed in the UCIN and maternity ward for scoring later. The footage was captured with the help of a collaborator who positioned the camera appropriately to record the assessment of each item of the evaluation. Participants could either be in an incubator (under intermediate or intensive care) or in the nursery. The first evaluation took place in loco and the second evaluation took place 14 days after the first, through the images assisted by evaluator 1 to prevent his memory from influencing the results. To achieve interexaminer reliability, another health professional with neonatal experience and training in occupational therapy was invited to participate in the research (evaluator 2).
- The stability of the instrument was assessed by test-retest reliability with 30 babies as a sample. The test-retest was performed by evaluator 1; however, between these evaluations, the 14-day interval was not respected because the neonates of the units (maternity and UCIN) could be discharged at any time, making it difficult for them to return to the data collection units. In addition, there was a great variability in the behavior of the newborn, exponentially changing the results of the evaluation. Thus, the second evaluation took place on the same day as the first with a sleep interval, breastfeeding, or a routine examination at the unit. The criterion used between the evaluations was to ensure that the behavioral state of the NB in the second evaluation was similar to the behavioral state as observed in the first evaluation.

### Data analysis

Data were analyzed using IBM SPSS Statistics for Windows version 22.0 (IBM Corp., Armonk, NY, USA). The participants received identification numbers to maintain anonymity and ensure blinding of the researcher responsible for the data analysis. Internal consistency was calculated using Cronbach alpha, which has recognized limits between 0.70 and 0.90. 15 The intra- and interexaminer reliability and reproducibility assessed through test-retest were calculated using the intraclass correlation coefficient (ICC 3.1). Each rater evaluated each subject, and the reliability was calculated from a single measurement. The ICC values interpretation considered correlations < 0.41 - weak, between 0.41 and 0.60 - moderate; between 0.61 and 0.80–strong or substantial, and between 0.81 and 1.00–almost perfect. 16

## RESULTS

After analyzing the translations from English to Portuguese (PV1 and PV2) of the 214 topics that structured the expanded instrument, > 80% agreement was found between the original and synthesized versions for 201 items. Eight items obtained 70% agreement, 4 obtained 60%, and only 1 item, which was the instrument's title, obtained agreement below 50%. Only 1 person agreed with the title (equivalent to 10% agreement); therefore, the title was changed.

In the equivalence assessment of the summarized HNNE, 25 items were evaluated, and 157 topics were translated and adapted to Brazilian culture. Nine specialists participated in this process, and there was an agreement of > 80% between the original and synthesized versions in 141 items. Thirteen items obtained 77.7% agreement, 1 obtained 66.6%, and 2 < 50%. Regarding the semantic validity, idiomatic validity, and conceptual validity, 3, 8, and 28 items, respectively, were changed.

The technical committee restructured the items with the lower-than-expected agreement, created the PCV, and subsequently sent it for backtranslation.

In stage 5, we sent the PCV, the RTCV, and the original version to the authors so that they could assess the equivalence of the versions and, if necessary, suggest some modifications. However, no changes were requested, or suggestions were added; thus, we retained the versions as presented. In this way, we consolidated each the final versions of each instrument (HNNE - Brazilian expanded and summarized versions [HNNE-Br]).

In phase 2 of reliability, 143 neonates participated in the study, with 98 (68.5%) full-term and 45 (31.5%) preterm babies. There were no extreme preterm births in this sample (gestational age [GA] < 28 weeks); however, 21 intermediate premature infants (GA between 28 and 34 weeks) and 24 late preterm infants (GA between 34 and 36 weeks) participated in the present study.

Unsatisfactory results were found regarding the internal consistency of each item, which was assessed using Cronbach alpha. These can be seen in Table 1 .

**Table 1:** Internal consistency by Hammersmith Neonatal Neurological Examination items

Item	Cronbach alpha α
Posture	0.555
Arm recoil	0.543
Arm traction	0.564
Leg recoil	0.537
Leg traction	0.554
Popliteal angle	0.527
Head control (1)	0.538
Head control (2)	0.525
Head lag	0.537
Ventral suspension	0.549
Flexor tone (1)	0.564
Flexor tone (2)	0.542
Leg extensor tone	0.588
Neck extensor tone	0.571
Increased extensor tone	0.557
Tendon reflex	0.542
Suck/gag	0.569
Palmar grasp	0.551
Plantar grasp	0.548
Placing	0.539
Moro reflex	0.546
Spontaneous movement (quantity)	0.515
Spontaneous movement (quality)	0.516
Head raising prone	0.553
Abnormal hand or toes postures	0.545
Tremor	0.549
Startle	0.539
Eye appearances	0.543
Auditory orientation	0.536
Visual orientation	0.541
Alertness	0.537
Irritability	0.530
Consolability	0.529
Cry	0.527

The interexaminer equivalence was performed by two different evaluators, with diverse academic backgrounds (physiotherapy and occupational therapy) in a subgroup formed by 30 newborns. The ICCs revealed high agreement between the evaluations in all domains ( Table 2 ).

**Table 2:** Interexaminer and intraexaminer reliability by intraclass correlation tests

	Interexaminer	Intraexaminer				
Domain	ICC	95%CI	*p-value*	ICC	95%CI	*-p-value*
Posture and tone	0.964	0.924–0.983	< 0.001	0.944	0.887–0.973	< 0.001
Tone patterns	0.969	0.934–0.985	< 0.001	1.000	–	< 0.001
Reflexes	0.951	0.897–0.977	< 0.001	0.962	0.923–0.982	< 0.001
Movements	0.972	0.942–0.987	< 0.001	0.993	0.986–0.997	< 0.001
Abnormal signs/patterns	0.912	0.816–0.958	< 0.001	0.990	0.979–0.995	< 0.001
Orientation and behavior	0.841	0.667–0.924	< 0.001	0.964	0.925–0.983	< 0.001
Total	0.964	0.925–0.983	< 0.001	0.955	0.908–0.978	< 0.001

Abbreviations: CI, confidence interval; ICC, intraclass correlation coefficient.

Intraexaminer reliability was performed in a subgroup of 30 newborns with a 14-day interval between onsite assessments and filming assessments, according to the interval proposed by Terwee et al. 17 It was found that there was a high level of agreement by domain between the evaluations, which can be seen in Table 2 .

The test-retest was applied to assess the measurement stability and calculate the ICC, which showed an excellent agreement between the domains, as shown in Table 3 .

**Table 3:** Test-retest reliability by intraclass correlation

Domain	ICC	95%CI	*p-value*
Posture and tone	0.996	0.991–0.998	< 0.001
Tone patterns	0.981	0.960–0.991	< 0.001
Reflexes	0.980	0.957–0.990	< 0.001
Movements	0.961	0.918–0.981	< 0.001
Abnormal signs/patterns	0.995	0.990–0.998	< 0.001
Orientation and behavior	0.854	0.693–0.930	< 0.001
Total	0.983	0.964–0.992	< 0.001

Abbreviations: CI, confidence interval; ICC, intraclass correlation coefficient.

## DISCUSSION

The cultural adaptation of the HNNE-Br instruments (expanded and summarized versions) to Brazilian Portuguese was carried out according to internationally recognized procedures, following the guidelines proposed by Ferrer et al., 12 Beaton et al. 10 and Guillemin et al. 18

The cultural adaptation process was initiated by translating the instruments into the Brazilian Portuguese language through two independent and bilingual translators, 10 with different academic backgrounds to increase the probability of finding more relevant terms. 19 According to Tanzer, 19 an adequate translation must promote linguistic, cultural, and contextual understanding so that the evaluated construct can maintain its integrality in both cultures. On the other hand, poorly translated instruments interfere negatively with data validity. 9

Subsequently, the technical committee compared the translated versions to develop a single and synthesized version, 20 which was sent to the expert committee, together with the original version to be evaluated regarding semantic, idiomatic, conceptual, cultural, and experiential discrepancies. A multidisciplinary committee of experts was essential to ensure the accuracy of the content. 21

Specialists must judge the relevance and range of the items of each instrument to assess the pertinence and scope, including face validity. 22 The experience of the experts is crucial to preserve the characteristics of the instrument that make it appropriate for the target population. 19 In this case, all suggestions made by the expert committee were rigorously examined and considered in the consensual versions of the instruments by obtaining satisfactory levels of agreement.

Ferrer et al. 12 point out that after obtaining the translations, they should be evaluated by a committee of experts and only then one can proceed with the backtranslation. Backtranslation is crucial because it allows authors to assess whether they maintain the same conceptual idea as the original instrument, thus preserving its construct. 20 The authors of the instrument agreed with the HNNE-Br versions presented, without any objection, which consolidated the final version without further changes.

The preliminary validation of the HNNE-Br, culturally adapted to Brazilian Portuguese, was carried out according to psychometric procedures recognized in the scientific literature by analyzing internal consistency and reproducibility. 20 23

In traditional psychometry, 10 respondents are recommended for each questionnaire item. In the expanded version of the HNNE, there are 34 items, while in the shorter one, there are 25 items. Following this concept, we would need to recruit between 250 and 340 participants. However, Sapnas et al. 24 demonstrated that subsamples with between 50 and 100 subjects are sufficient to analyze the psychometric properties, especially social constructs. They believed that 10 respondents per item are more than necessary and guaranteed that 100 subjects are sufficient to verify the initial psychometric properties of an instrument being tested in another population, allowing a desirable conclusion to be reached. 24 Therefore, the sample size of the present study was adequate.

Cronbach alpha has recognized limits between 0.70 and 0.90, with values < 0.70 indicating nonconsistency. 25 Although the HNNE-Br Cronbach's alpha coefficient values are below what are considered acceptable, studies on the psychometric properties of the HNNE in its original version or other versions have not been found, making a comparison impossible. Keszei et al. 26 reported that low internal consistency values can mean that the items measure different attributes. It happens on the HNNE because this instrument evaluates the motor, visual, and auditory systems, and a baby with CP may have an impaired motor system while the other systems may remain intact, corroborating the lack of consistency between the items.

The inter-rater reliability, performed in a subgroup formed by 30 NBs through the initial filmed evaluations, showed high agreement, agreeing with the results provided by the authors when developing the instrument 27 and by Eeles et al., 28 who found excellent reliability (ICC > 0.74) for the total score when evaluating premature NBs.

Evaluating through filming is widely used in the literature for intrarater reliability, being considered by many to be a satisfactory method. 29 30 However, some researchers highlight difficulties in evaluating individuals from videos, as filming limits viewing from different angles. 31 32 Thus, we invited one occupational therapist, who worked in the intermediate care unit, to carry out the filming, to capture the details of the evaluation, and highlight the response of the NB after a stimulus, such as the reflex. This form of assessment allows no changes between the behavior or response of the individual, which could be different if the reassessment occurred after 14 days, thus, compromising data reliability.

As with previous measurements, the period between the test and retest is a factor that must be considered. If carried out in too short a period, the results may be contaminated by the memory effect of the first application. On the other hand, if carried out after a long period, results are susceptible to changes and the acquisition of new skills and may compromise the interpretation of the obtained reliability coefficient. 26

The test-retest evaluations took place on the same day because the acquisitions and behaviors of the neonates were quite variable. The results showed almost perfect agreement in nearly all items, with the lowest values attributed to the behavior domain (which may vary if the newborn is hungry, sleepy, colic, or even due to the high degree of handling in the sector for collections of evaluations). The highest values were for posture and tone, which, despite being variable, did not change much on the same day.

In conclusion, the analyzed properties suggest that the HNNE is adapted and reliable for the Brazilian population and can be a useful instrument for the neurological assessment of Brazilian newborns, to identify changes in neurological development, and to refer them early to the stimulation or early rehabilitation units. The HNNE is an easy and fast application tool, has open access, and, therefore, is a promising option to be used in the context of primary care in Brazil.

The availability of an instrument with these characteristics will favor the inclusion of child development surveillance in the daily lives of health professionals, enabling intervention at an appropriate time in cases suspected of alterations. In addition, using a standardized and reliable instrument for Brazil will also allow the generation of epidemiological information to promote public policies and to compare with studies carried out in other countries.
