# Erratum

**DOI:** 10.1055/s-0045-1802550

**Published:** 2025-02-10

In the article “Cultural adaptation and reliability assessment of the Hammersmith neonatal neurological examination for Brazilian newborns at risk of cerebral palsy”, published in Arq. Neuropsiquiatr. 2023;81(1): 47–54 under DOI 10.1055/s-0042-1758863 ,

**Where it reads:**

To perform a cross-cultural adaptation and reliability assessment of a Brazilian version of the Hammersmith Neonatal Neurological Examination (HNNE), expanded and summarized.

**It should be:**

To perform a cross-cultural adaptation and reliability assessment of a Brazilian version of the Hammersmith Neonatal Neurological Examination (HNNE), full original and summarized.

**Where it reads:**

A methodological, cross-sectional, nonexperimental quantitative analysis was conducted in two phases as follows: cultural adaptation of the HNNE, expanded and summarized, and reliability assessment of the Brazilian version of the HNNE.

**It should be:**

A methodological, cross-sectional, nonexperimental quantitative analysis was conducted in two phases as follows: cultural adaptation of the HNNE, full original and summarized (short), and reliability assessment of the Brazilian version of the HNNE.

**Where it reads:**

The Brazilian HNNE expanded and summarized versions can be considered to be adapted and reliable for the neurological assessment of Brazilian newborns to identify changes in neurological development and early referral to the stimulation or early rehabilitation units and as a promising option to be used in the context of primary care in Brazil.

**It should be:**

The Brazilian HNNE full and short versions can be considered to be adapted and reliable for the neurological assessment of Brazilian newborns to identify changes in neurological development and early referral to the stimulation or early rehabilitation units and as a promising option to be used in the context of primary care in Brazil.

**Where it reads:**

Foi realizada análise quantitativa metodológica, transversal e não experimental em duas fases: adaptação cultural do HNNE, ampliada e resumida, e avaliação da confiabilidade da versão brasileira do HNNE. A primeira fase foi desenvolvida em cinco etapas (tradução inicial, síntese da tradução, comitê de especialistas, retrotradução e submissão ao autor), sendo avaliadas as questões semânticas, conteúdo e validade de face.

**It should be:**

Foi realizada análise quantitativa metodológica, transversal e não experimental em duas fases: adaptação cultural do HNNE, original completo e resumido (curto), e avaliação da confiabilidade da versão brasileira do HNNE. A primeira fase foi desenvolvida em cinco etapas (tradução inicial, síntese da tradução, comitê de especialistas, retrotradução e submissão ao autor), sendo avaliadas as questões semânticas, conteúdo e validade de face.

**Where it reads:**

The Hammersmith Neurological Examination has two versions: the expanded and summarized versions of the Hammersmith Neonatal Neurological Examination (HNNE), which evaluates newborns (NBs) up to 3 months old, and the Hammersmith Infant Neurological Examination (HINE), which evaluates infants between 30 days and 24 months old.

**It should be:**

The Hammersmith Neurological Examination has two versions: full and short versions of the Hammersmith Neonatal Neurological Examination (HNNE), which evaluates newborns (NBs) up to 3 months old, and the Hammersmith Infant Neurological Examination (HINE), which evaluates infants between 30 days and 24 months old.

**Where it reads:**

The expanded version of the HNNE consists of 34 items subdivided into 6 categories as follows: 1) posture and tone, 2) tone patterns, 3) reflexes, 4) movements, 5) abnormal signs, and 6) orientation and behavior.

**It should be:**

The full version of the HNNE consists of 34 items subdivided into 6 categories as follows: 1) posture and tone, 2) tone patterns, 3) reflexes, 4) movements, 5) abnormal signs, and 6) orientation and behavior.

**Where it reads:**

In 2005, a short and simplified version of the HNNE was prepared for screening, which consisted of 25 items under the following categories: posture and tone, movements, reflexes, guidance, and behavior. ^5^

**It should be:**

A short and simplified version of the HNNE was prepared for screening, which consisted of 25 items under the following categories: posture and tone, movements, reflexes, guidance, and behavior. ^6^

**Where it reads:**

Both versions can be applied to premature infants.

**It should be:**

Both versions can be applied to premature infants, respecting corrected age for application in preterm babies.

**Where it reads:**

This way, we decided to perform a cross-cultural adaptation and assess the reliability of a Brazilian version of the HNNE (expanded and summarized).

**It should be:**

This way, we decided to perform a cross-cultural adaptation and assess the reliability of a Brazilian version of the HNNE (full and short).

**Where it reads:**

The present methodological, cross-sectional, nonexperimental study, approved by the ethics committee under opinion No. 1,809,858,was conducted in 2 phases as follows: cultural adaptation of the HNNE (expanded and summarized) and reliability assessment of the Brazilian version of the HNNE (expanded).

**It should be:**

The present methodological, cross-sectional, nonexperimental study, approved by the ethics committee under opinion No. 1,809,858,was conducted in 2 phases as follows: cultural adaptation of the HNNE (full and short) and reliability assessment of the Brazilian version of the HNNE (full).

**Where it reads:**

Cultural adaptation of the HNNE – expanded and summarized ^6^ to Brazilian neonates.

**It should be:**

Cultural adaptation of the HNNE – full and short ^6^ to Brazilian neonates.

**Where it reads:**

We obtained authorization from the authors of the instrument via email to carry out the HNNE translation, cultural adaptation, and psychometric validation of the expanded and summarized versions.

**It should be:**

We obtained authorization from the authors of the instrument via email to carry out the HNNE translation, cultural adaptation, and psychometric validation of the full and short versions.

**Where it reads:**

Two Brazilian translators (T1 and T2) independently translated the original version of the HNNE (expanded and summarized).

**It should be:**

Two Brazilian translators (T1 and T2) independently translated the original version of the HNNE (full and short).

**Where it reads:**

We analyzed the internal consistency, stability, and equivalence to verify the reliability of the HNNE-Br (expanded). The 25 items of the HNNE-Br summarized are present in the expanded version, so the results are the same for both versions:

**It should be:**

We analyzed the internal consistency, stability, and equivalence to verify the reliability of the HNNE-Br (full version). The 25 items of the HNNE-Br short are present in the full original version, so the results are the same for both versions:

**Where it reads:**

The VCP and VCRT versions were sent to the author of the instrument and the team who approved the versions consolidating the final version (HNNE-Br) of the HNNE instrument in all their versions (HNNE expanded and summarized + HINE) for the Brazilian population. It is important to note that the authors did not make suggestions or indicate changes to be made in the documents sent to them.

**It should be:**

The VCP and VCRT versions were sent to the author of the instrument and the team who approved the versions consolidating the final version (HNNE-Br) of the HNNE instrument in all their versions (HNNE full and short + HINE) for the Brazilian population. It is important to note that the authors did not make suggestions or indicate changes to be made in the documents sent to them.

**Where it reads:**

The expanded HNNE had 34 items, each with 5 possible answers; thus, in the stages of cultural adaptation, it was broken down into 170 topics of analysis, in addition to items that included comments, observations, characterization of the evaluated items, and a summary of the instrument, totaling 214 topics translated and adapted to the Brazilian culture.

**It should be:**

The full HNNE had 34 items, each with 5 possible answers; thus, in the stages of cultural adaptation, it was broken down into 170 topics of analysis, in addition to items that included comments, observations, characterization of the evaluated items, and a summary of the instrument, totaling 214 topics translated and adapted to the Brazilian culture.

**Where it reads:**

Reliability of the Brazilian version of the HNNE (HNNE-Br) – expanded and summarized.

**It should be:**

Reliability of the Brazilian version of the HNNE (HNNE-Br) – full and short.

**Where it reads:**

The Cronbach alpha test analyzed the internal consistency14 of the HNNE-Br (expanded) and was performed witha total sample of 143 NBs. The evaluations took place as near as possible after birth, and some NBs were evaluated a few hours after birth, while others a few days later.

**It should be:**

The Cronbach alpha test analyzed the internal consistency14 of the HNNE-Br (full version) and was performed witha total sample of 143 NBs. The evaluations took place as near as possible after birth, and some NBs were evaluated a few hours after birth, while others a few days later.

**Where it reads:**

After analyzing the translations from English to Portuguese (PV1 and PV2) of the 214 topics that structured the expanded instrument, >80% agreement was found between the original and synthesized versions for 201 items.

**It should be:**

After analyzing the translations from English to Portuguese (PV1 and PV2) of the 214 topics that structured the full version of the instrument, >80% agreement was found between the original and synthesized versions for 201 items.

**Where it reads:**

In this way, we consolidated each the final versions of each instrument (HNNE-Brazilian expanded andsummarized versions [HNNEBr]).

**It should be:**

In this way, we consolidated each the final versions of each instrument (HNNE-Brazilian full and short versions [HNNEBr]).

**Where it reads:**

The cultural adaptation of the HNNE-Br instruments (expanded and summarized versions) to Brazilian Portuguese was carried out according to internationally recognized procedures, following the guidelines proposed by Ferrer et al., ^12^ Beaton et al. ^10^ and Guillemin et al. ^18^

**It should be:**

The cultural adaptation of the HNNE-Br instruments (full and short versions) to Brazilian Portuguese was carried out according to internationally recognized procedures, following the guidelines proposed by Ferrer et al., ^12^ Beaton et al. ^10^ and Guillemin et al. ^18^

**Where it reads:**

In the expanded version of the HNNE, there are 34 items, while in the shorter one, there are 25 items.

**It should be:**

In the full version of the HNNE, there are 34 items, while in the shorter one, there are 25 items.

